# Correlation between fundus autofluorescence and visual function in patients with cone-rod dystrophy

**DOI:** 10.1038/s41598-021-81597-7

**Published:** 2021-01-21

**Authors:** Satoru Kanda, Takumi Hara, Ryosuke Fujino, Keiko Azuma, Hirotsugu Soga, Ryo Asaoka, Ryo Obata, Tatsuya Inoue

**Affiliations:** 1 Asahi General Hospital, 1326, Asahi-shi, Chiba, Japan; 2 Department of Ophthalmology, The University of Tokyo, 7-3-1 Hongo, Bunkyo-ku, Tokyo, Japan; 3 Department of Ophthalmology, Seirei Hamamatsu General Hospital, Shizuoka, Japan; 4 Seirei Christopher University, Shizuoka, Japan; 5 Department of Ophthalmology and Micro-Technology, Yokohama City University, 4-57 Urafune-cho, Minami-ku, Yokohama, Kanagawa 232-0024 Japan

**Keywords:** Macular degeneration, Retinal diseases

## Abstract

This study aimed to investigate the relationship between autofluorescence (AF) signal measured with ultra-wide field imaging and visual functions in patients with cone-rod dystrophy (CORD). A retrospective chart review was performed for CORD patients. We performed the visual field test and fundus autofluorescence (FAF) measurement and visualized retinal structures with optical coherence tomography (OCT) on the same day. Using binarised FAF images, we identified a low FAF area ratio (LFAR: low FAF/30°). Relationships between age and logMAR visual acuity (VA), central retinal thickness (CRT), central choroidal thickness (CCT), mean deviation (MD) value, and LFAR were investigated. Thirty-seven eyes of 21 CORD patients (8 men and 13 women) were enrolled. The mean patient age was 49.8 years. LogMAR VA and MD were 0.52 ± 0.47 and − 17.91 ± 10.59 dB, respectively. There was a significant relationship between logMAR VA and MD (*p* = 0.001). LogMAR VA significantly correlated with CRT (*p* = 0.006) but not with other parameters. Conversely, univariate analysis suggested a significant relationship between MD and LFAR (*p* = 0.001). In the multivariate analysis, LFAR was significantly associated with MD (*p* = 0.002). In conclusion, it is useful to measure the low FAF area in patients with CORD. The AF measurement reflects the visual field deterioration but not VA in CORD.

## Introduction

Retinal dystrophy belongs to a set of hereditary diseases that affect photoreceptors and lead to blindness in developed countries^1^. The worldwide prevalence of retinal dystrophy is estimated at more than 2 million patients, and several causative genes have been identified^2^. In inherited retinal dystrophies, cone-rod dystrophy (CORD) is characterized by primary cone photoreceptor impairment^3^. At the end stage, CORDs occasionally cause peripheral visual dysfunction, followed by the loss of rod photoreceptor cells.

Fundus autofluorescence (FAF) allows us to evaluate retinal pigment epithelium (RPE) and photoreceptor function non-invasively. The usefulness of FAF has been reported in several retinal diseases, such as retinitis pigmentosa (RP), Stargardt disease, and CORD, in previous studies^4–16^. In general, the hyperfluorescence in blue FAF shows abnormal accumulation of lipofuscin and suggests degenerating photoreceptor cells; in contrast, the hypofluorescence in blue FAF is considered to be RPE and photoreceptor cell atrophy^17–21^.

The Optos (Optos 200Tx; Optos, Dunfermline, UK) allows the visualization in the retina at 200° in a single frame. In previous studies, the utility of this instrument was revealed in RP^12,22^, age-related macular degeneration^23^, chorioretinitis^24^, and retinal detachment^25^. FAF can also be measured in this area using the device. The aim of our study was to investigate the relationship between FAF measured with the Optos and visual functions in patients with CORD.

## Methods

The present study enrolled consecutive CORD patients. The study protocol was approved by the Research Ethics Committee of the Graduate School of Medicine and Faculty of Medicine at The University of Tokyo. The study adhered to the tenets of the Declaration of Helsinki. Written informed consent was obtained from each participant. Written informed consent and consent to publish the data in an online open access publication were obtained from each participant. For the participant under the age of 20, informed consent was obtained from the parents.

The diagnosis of CORD was made based on optical coherence tomography (OCT), fluorescein angiography (FA), and electroretinogram (ERG). Each patient underwent visual field testing with the Humphrey field analyser (HFA; Carl Zeiss Meditec, Dublin, CA) using the Swedish interactive threshold algorithm standard programme and the 10-2 test pattern. Unreliable visual fields were excluded from the study; the fixation losses ≥ 20%, the false-positive rates ≥ 15%, and the false-negative rates ≥ 33% as determined by SITA-Standard^26^.

In addition to visual field testing with HFA, wide-field fundus FAF images were obtained using the Optos imaging system simultaneously. The Optos uses green light of a wavelength of 532 nm for excitation and detects the emitted signal with a detector for light of a wavelength of 570–780 nm^27^. To improve image quality and because OCT measurements and ophthalmoscopic examination were concurrently performed, the pupil was dilated using topical applications of tropicamide and phenylephrine. We simultaneously obtained spectral-domain OCT (SD-OCT) images using Spectralis OCT (Heidelberg Engineering, Heidelberg, Germany). Subsequently, the central retinal thickness (CRT) and central choroidal thickness (CCT) were measured in each CORD eye.

**Figure 1:**
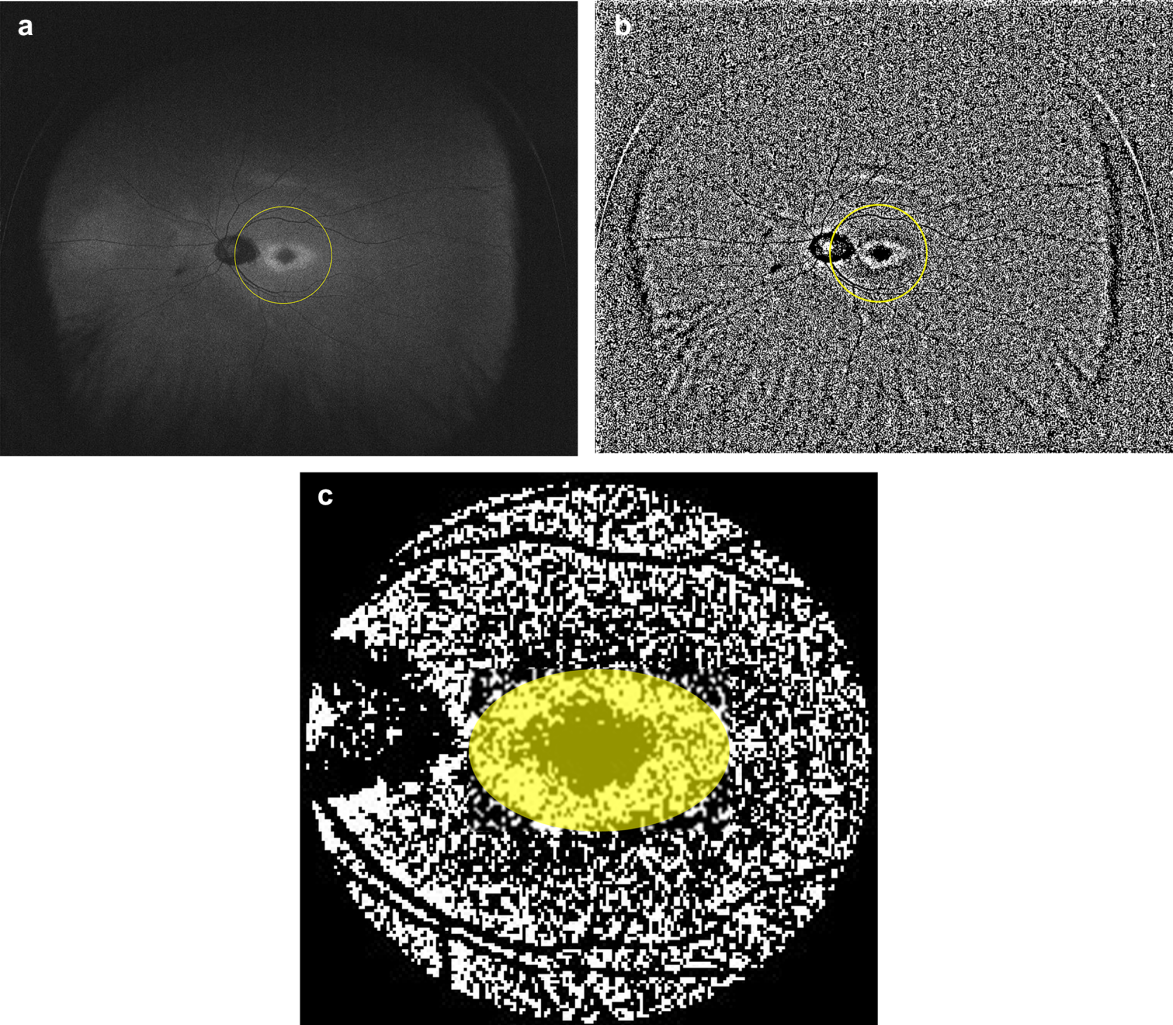
(**a**) Example of a fundus autofluorescence image of a 61-year-old man with cone-rod dystrophy. The yellow circle indicates the area within 30° around the macula. *FAF* fundus autofluorescence, *CORD* cone-rod dystrophy. (**b**) Binarization of the fundus autofluorescence image. The binarised image in the yellow circle area was used to calculate the low FAF area ratio. *FAF* Fundus autofluorescence. (**c**) Calculation of the low fundus autofluorescence (FAF) area ratio (LFAR). Using the binarised image, the low FAF signal was measured as the sum of black pixels in the presumable abnormal autofluorescence area (yellow circle). Next, LFAR was calculated as the ratio of black pixels in the abnormal FAF area to total pixels within 30° around the macula.

Using the images measured with Optos, we calculated a low FAF area using the ImageJ software (http://imagej.nih.gov/ij/; provided in the public domain by the National Institutes of Health, Bethesda, MD, USA). We analysed FAF images within 30° in the macula because the centre of the optic nerve head is located approximately 15° nasal to the fovea (Fig. 1A). First, the border of the abnormal FAF area was identified. Next, the obtained FAF images were binarised with Niblack's method (Fig. 1B). The images were converted to 8-bit images and adjusted with Niblack's auto-local threshold in ImageJ software. With Niblack's method, we could automatically separate the low FAF area from the total abnormal FAF area. Finally, the sum of low FAF signals was measured within the abnormal autofluorescence area in the binarised image, and the low FAF area ratio (LFAR) was calculated as the sum of low FAF pixels within 30° (Fig. 1C).

Correlations between visual functions (logMAR VA and the MD value) and morphologic parameters (CRT, CCT, and LFAR) were investigated using the linear mixed model. Subsequently, models were selected to identify the optimal linear regression model using the second-order bias-corrected Akaike's information criterion (AICc) index from all 2^4^ patterns, comprising four variables (age, LFAR, CRT, and CCT). AIC is a well-known statistical measure used in model selection, and AICc represents a corrected version of AIC that provides an accurate estimate even with a small sample size^28,29^.

Variables selected through model selection were considered to be statistically significant. All statistical analyses were performed using the statistical programming language R ver. 3.4.3 (The R Foundation for Statistical Computing, Vienna, Austria).

## Results

**Figure 2:**
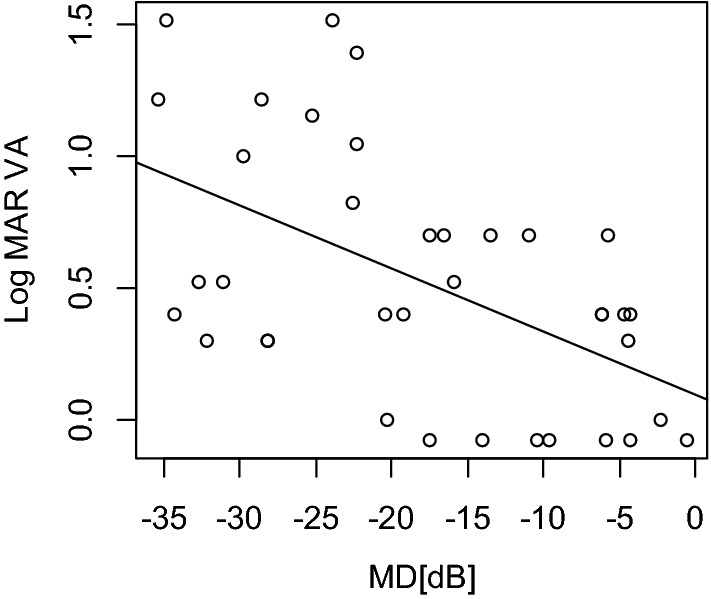
Relationship between logMAR VA and the MD value. There was a significant correlation between logMAR VA and MD (*p* = 0.001, linear mixed model). *VA* visual acuity, *MD* mean deviation.

**Figure 3:**
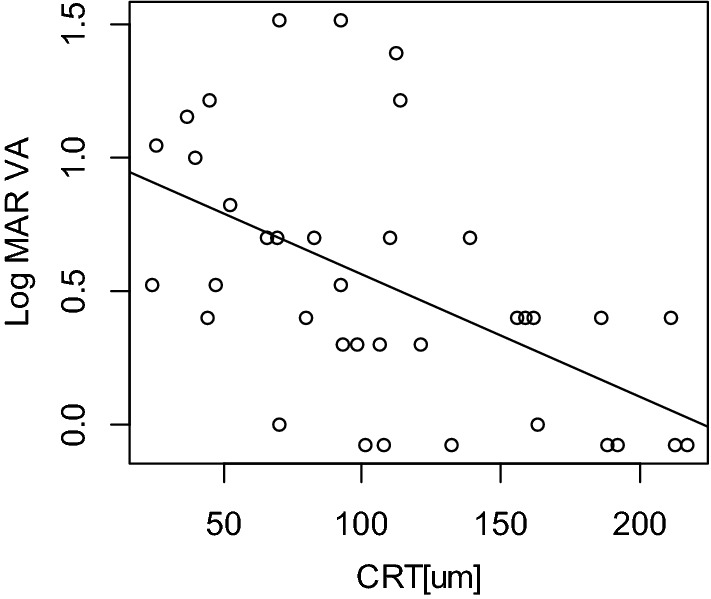
Relationship between logMAR visual acuity (VA) and central retinal thickness (CRT). CRT was significantly associated with logMAR VA (*p* = 0.006, linear mixed model). *CRT* central retinal thickness, *logMAR VA* logarithm minimal angle resolution visual acuity.

Characteristics of the subjects are shown in Table 1. Thirty-seven eyes (18 right and 19 left eyes) of 21 patients with CORD (eight men and 13 women) were expand. LogMAR VA and MD were 0.52 ± 0.47 [− 0.079 to 1.52] and − 17.91 ± 10.59 [− 35.45 to − 0.58] dB, respectively. There was a significant relationship between logMAR VA and the MD value (Fig. 2*p* = 0.001, linear mixed model). The univariate analysis suggested that logMAR VA significantly correlated with CRT (Fig. 3, *p* = 0.006, linear mixed model) but not with other parameters, i.e., age, LFAR, or CCT (*p* > 0.05). Based on the AICc model selection, among age, LFAR, CRT, and CCT, the optimal model for logMAR VA included only CRT (AICc = 39.3); LogMAR VA = 0.98–0.0040 (standard error [SE] = 0.00136, *p* = 0.006) × CRT (Table 2).

**Table 1:** Characteristics.

Variable	Mean ± SD	Range
Gender (male:female)	8:13	
Age (years)	49.4 ± 15.6	17 to73
LogMAR VA	0.52 ± 0.47	− 0.079 to 1.52
Mean deviation	− 17.9 ± 10.6	− 35.45 to − 0.58
LFAR	0.11 ± 0.17	0.0050 to 0.74
CRT (μm)	108.6 ± 55.1	24 to 217
CCT (μm)	206.4 ± 75.7	104 to 453
FL	0.047 ± 0.061	0 to 0.19
FP (%)	1.49 ± 2.26	0 to 10
FN (%)	4.03 ± 6.09	0 to 25

*SD* standard deviation, *Log MAR* logarithm minimal angle resolution, *LFAR* low fundus autofluorescence area ratio, *CRT* central retinal thickness, *CCT* central choroidal thickness, *FL* fixation loss, *FP* false positive, *FN* false negative.

**Table 2:** Relationship between logMAR VA and other parameters.

Variable	Univariate analysis			Multivariate analysis		
	Estimate	Standard error	p value	Estimate	Standard error	p value
Age	0.00029	0.0066	0.97	N.S	N.S	N.S
LFAR	0.47	0.64	0.47	N.S	N.S	N.S
CRT	0.0040	0.0014	0.006	− 0.0040	0.0014	0.006
CCT	0.0016	0.0011	0.12	N.S	N.S	N.S

logMAR VA, logarithm minimal angle resolution visual acuity; LFAR, low fundus autofluorescence area ratio; CRT, central retinal thickness; CCT, central choroidal thickness; N. S., not selected.

**Figure 4:**
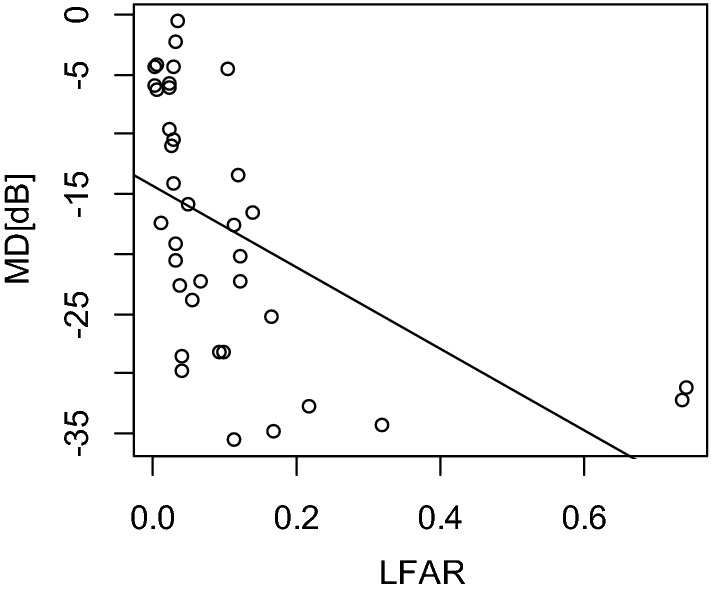
Relationship between the mean deviation (MD) and low fundus autofluorescence area ratio (LFAR). There was a significant relationship between MD and LFAR (*p* = 0.006, linear mixed model). *MD* mean deviation, *LFAR* low fundus autofluorescence area ratio.

The univariate analysis revealed a significant correlation between MD and LFAR (Fig. 4, *p* = 0.001, linear mixed model) but not between MD and other parameters (age, CRT, and CCT, *p* > 0.05). The optimal model for MD included only LFAR (AICc = 232.4). MD = − 14.9 to 34.7 (SE = 9.71, *p* = 0.002) × LFAR. No remaining variable, i.e., age, CCT, or CRT, was included (Table 3).

**Table 3:** Relationship between MD value and other parameters.

Variable	Univariate analysis			Multivariate analysis		
	Estimate	Standard error	p value	Estimate	Standard error	p value
Age	− 0.014	0.15	0.92	N.S	N.S	N.S
LFAR	− 34.7	9.8	0.001	− 34.7	9.8	0.001
CRT	0.024	0.017	0.17	N.S	N.S	N.S
CCT	0.018	0.011	0.11	N.S	N.S	N.S

*MD* mean deviation, *LFAR* low fundus autofluorescence area ratio, *CRT* central retinal thickness, *CCT* central choroidal thickness, *N.S*. not selected.

## Discussion

In the current study, the visual field and OCT measurements were performed along with wide-field FAF in patients with CORD. We found that CRT significantly correlated with logMAR VA, suggesting that CRT was the most useful parameter to predict logMAR VA. LFAR correlated with MD, but CRT did not correlate with MD. This implied that the FAF measurement was more useful compared to OCT parameters when analysing visual field deterioration in CORD.

In daily practice with CORD, VA is the most frequently used method to evaluate visual function. The current result that VA was significantly associated with CRT supported the usefulness of this approach. However, VA mainly reflected the visual function around the macula, and more detailed evaluation of retinal sensitivity could not be performed without measuring the visual field. The current results suggested that MD of the visual field correlated with LFAR but not with CRT or CCT. This result was in agreement with that of a previous study by Oishi et al., who suggested that there was a correlation between the area of abnormal FAF and the central scotoma size measured with Goldmann perimetry but not between the area of abnormal FAF and logMAR VA^30^. These findings suggested that the disease status of CORD could not be completely explained simply based on the thicknesses of the retina and choroid and were instead useful to evaluate retinal atrophy, particularly in the RPE and photoreceptor cells, with the FAF measurement. However, we did not evaluate the outer retinal structure, such as ellipsoid zone and interdigitation zone, which were reported to be affected in eyes with CORD^31^. It appears that the outer retinal structure rather than CRT might be associated with visual field deterioration.

There are previous reports that suggested the usefulness of measuring the FAF pattern in eyes with CORD. In contrast to low AF resulting from retinal atrophy, particularly in the RPE and photoreceptor cells, the hyper-AF area suggested a degenerative process. The hyperfluorescent AF signal was also important when assessing the disease status of CORD^32,33^. For instance, peripherin-2 (PRPH2) is one of the causative genes of CORD, and the mutation of PRPH2 has been shown to be associated with a speckled pattern of FAF^34^. Other studies reported that FAF imaging in eyes with X-linked RPGR-associated CORD demonstrated parafoveal hyperfluorescent rings, and the size of the rings gradually increased over time^16,35^. Furthermore, hyper-AF was observed in the abnormal area in eyes with RP^36^. Leber congenital amaurosis caused by the *Crumb1* gene demonstrates a unique AF pattern in which the AF signal is preserved in the para-arteriolar region^37^. In the current study, to highlight the usefulness of the hypo-AF signal, we calculated LFAR in eyes with CORD even when both hyper- and hypo-AF signals were observed in the abnormal AF area (Fig. 1). It is of our further interest to examine whether similar results are obtained when the abnormal hyperfluorescent area are also analyzed. Furthermore, genetic backgrounds of CORD were not investigated in the current study, so further studies are required to clarify the relationship between an abnormal FAF area and VF deterioration considering genetic information.

A limitation of the current study was the accuracy of the visual field test. We used HFA to evaluate the visual function in eyes with CORD. However, severe loss of central visual function might hamper accurate visual field tests performed with HFA because of poor fixation. It may be valid to measure the visual field using a microperimeter, in which the position of the retina is tracked, and the same location is stimulated at each target presentation even with the loss of central visual function. Moreover, the sample size was relatively small. A further study involving a larger number of eyes would be required.

## Conclusion

In conclusion, it is useful to measure the low FAF area in patients with CORD, as suggested by the significant relationship between LFAR and MD.
